# Correction: Tracking nucleic acid nanocapsule assembly, cellular uptake and disassembly using a novel fluorescently labeled surfactant

**DOI:** 10.1039/d5ra90055g

**Published:** 2025-05-14

**Authors:** Md Arifuzzaman, Alyssa K. Hartmann, Jessica L. Rouge

**Affiliations:** a Department of Chemistry, University of Connecticut 55 North Eagleville Road Storrs CT 06269 USA jessica.rouge@uconn.edu

## Abstract

Correction for ‘Tracking nucleic acid nanocapsule assembly, cellular uptake and disassembly using a novel fluorescently labeled surfactant’ by Md Arifuzzaman *et al.*, *RSC Adv.*, 2020, **10**, 42349–42353, https://doi.org/10.1039/D0RA09472B.

The authors regret the omission of a funding acknowledgement in the original article. This acknowledgement is given below.

“We also acknowledge support from the NIH grant R35GM138226-02”.

The Royal Society of Chemistry apologises for these errors and any consequent inconvenience to authors and readers.

